# Effects of canagliflozin, a sodium glucose co-transporter 2 inhibitor, on blood pressure and markers of arterial stiffness in patients with type 2 diabetes mellitus: a post hoc analysis

**DOI:** 10.1186/s12933-017-0511-0

**Published:** 2017-02-27

**Authors:** Michael Pfeifer, Raymond R. Townsend, Michael J. Davies, Ujjwala Vijapurkar, Jimmy Ren

**Affiliations:** 1 Janssen Scientific Affairs, LLC, 1125 Trenton-Harbourton Road, Titusville, NJ 08560 USA; 2 Perelman School of Medicine, University of Pennsylvania, 122 Founders Building, 3400 Spruce Street, Philadelphia, PA 19104 USA; 3 Janssen Research & Development, LLC, 920 US Highway 202 South, Raritan, NJ 08869 USA

**Keywords:** Canagliflozin, Sodium glucose co-transporter 2 (SGLT2) inhibitor, Type 2 diabetes, Blood pressure, Ambulatory blood pressure monitoring, Pulse pressure, Mean arterial pressure, Double product

## Abstract

**Background:**

Physiologic determinants, such as pulse pressure [difference between systolic blood pressure (SBP) and diastolic BP (DBP)], mean arterial pressure (2/3 DBP + 1/3 SBP), and double product [beats per minute (bpm) × SBP], are linked to cardiovascular outcomes. The effects of canagliflozin, a sodium glucose co-transporter 2 (SGLT2) inhibitor, on pulse pressure, mean arterial pressure, and double product were assessed in patients with type 2 diabetes mellitus (T2DM).

**Methods:**

This post hoc analysis was based on pooled data from four 26-week, randomized, double-blind, placebo-controlled studies evaluating canagliflozin in patients with T2DM (N = 2313) and a 6-week, randomized, double-blind, placebo-controlled, ambulatory BP monitoring (ABPM) study evaluating canagliflozin in patients with T2DM and hypertension (N = 169). Changes from baseline in SBP, DBP, pulse pressure, mean arterial pressure, and double product were assessed using seated BP measurements (pooled studies) or averaged 24-h BP assessments (ABPM study). Safety was assessed based on adverse event reports.

**Results:**

In the pooled studies, canagliflozin 100 and 300 mg reduced SBP (−4.3 and −5.0 vs −0.3 mmHg) and DBP (−2.5 and −2.4 vs −0.6 mmHg) versus placebo at week 26. Reductions in pulse pressure (−1.8 and −2.6 vs 0.2 mmHg), mean arterial pressure (−3.1 and −3.3 vs −0.5 mmHg), and double product (−381 and −416 vs −30 bpm × mmHg) were also seen with canagliflozin 100 and 300 mg versus placebo. In the ABPM study, canagliflozin 100 and 300 mg reduced mean 24-h SBP (−4.5 and −6.2 vs −1.2 mmHg) and DBP (−2.2 and −3.2 vs −0.3 mmHg) versus placebo at week 6. Canagliflozin 300 mg provided reductions in pulse pressure (−3.3 vs −0.8 mmHg) and mean arterial pressure (−4.2 vs −0.6 mmHg) compared with placebo, while canagliflozin 100 mg had more modest effects on these parameters. Canagliflozin was generally well tolerated in both study populations.

**Conclusions:**

Canagliflozin improved all three cardiovascular physiologic markers, consistent with the hypothesis that canagliflozin may have beneficial effects on some cardiovascular outcomes in patients with T2DM.

*Trial registration* ClinicalTrials.gov Identifier: NCT01081834 (registered March 2010); NCT01106677 (registered April 2010); NCT01106625 (registered April 2010); NCT01106690 (registered April 2010); NCT01939496 (registered September 2013)

## Background

Type 2 diabetes mellitus (T2DM) is associated with increased cardiovascular morbidity and mortality [1]. T2DM has been shown to be an independent risk factor for cardiovascular disease, and common comorbidities of T2DM, such as dyslipidemia and hypertension, can further increase this risk [1, 2]. It is estimated that patients with T2DM and hypertension have a fourfold higher risk of developing cardiovascular disease compared with healthy individuals [2]. Furthermore, data from the Framingham Heart Study showed that patients with hypertension at the time of T2DM diagnosis had significantly higher rates of all-cause mortality and cardiovascular events compared with normotensive patients with T2DM [3]. The importance of effective blood pressure (BP) control to prevent cardiovascular events in patients with T2DM has been well established [1, 2]. Current guidelines recommend a target BP of <140/90 mmHg for most patients with T2DM and hypertension to reduce the risk of cardiovascular events, such as congestive heart failure and stroke [1, 4].

Arterial stiffness is also an established risk factor of cardiovascular events and mortality [5–7]. In particular, the physiologic determinants, such as pulse pressure {a surrogate for pulse wave velocity used to assess arterial stiffness [difference between systolic BP (SBP) and diastolic BP (DBP)]}, mean arterial pressure [measure of cardiac output, systemic vascular resistance, and central venous pressure (2/3 DBP + 1/3 SBP)], and double product [a measure of cardiac workload and myocardial oxygen demand (heart rate × SBP)], have been linked to cardiovascular outcomes and may provide additional information for predicting cardiovascular disease risk [8, 9]. Therefore, assessment of these surrogate BP parameters may be particularly relevant to assess cardiac efficiency in patients with T2DM.

Canagliflozin is a sodium glucose co-transporter 2 (SGLT2) inhibitor approved to treat adults with T2DM [10]. SGLT2 inhibition has been shown to increase urinary glucose excretion (UGE), resulting in decreased plasma glucose levels in patients with T2DM [11–13]. Increased UGE also results in mild osmotic diuresis and a net caloric loss that contributes to reductions in body weight and BP [12, 14]. Across Phase 3 studies ranging from 26 to 104 weeks in duration, canagliflozin provided improvements in glycemic control and reductions in body weight and BP, with a favorable safety and tolerability profile, in a broad range of patients with T2DM as monotherapy or in combination with other antihyperglycemic agents (AHAs) [15].

In a pooled analysis of four placebo-controlled, Phase 3 studies, canagliflozin treatment was associated with reductions in SBP at 26 weeks in both the overall population of patients with T2DM and a subgroup of patients with elevated SBP at baseline (≥140 mmHg) [16]. A separate randomized, double-blind, placebo-controlled study assessing the immediate effects of canagliflozin on BP using ambulatory BP monitoring (ABPM) reported reductions in mean 24-h SBP after 6 weeks of canagliflozin treatment in patients with T2DM and hypertension [17]. Using pooled data from the four 26-week, placebo-controlled studies in a general population of patients with T2DM and data from the 6-week ABPM study in patients with T2DM and hypertension, this post hoc analysis assessed the immediate and longer-term changes in pulse pressure, mean arterial pressure, and double product with canagliflozin treatment.

## Methods

### Study design and patient populations

This post hoc analysis was based on data from two study populations: a pooled population consisting of four 26-week, randomized, double-blind, placebo-controlled, Phase 3 studies evaluating canagliflozin in patients with T2DM (N = 2313) and a 6-week, randomized, double-blind, placebo-controlled study evaluating canagliflozin in patients with T2DM and hypertension (N = 169).

The four placebo-controlled studies included assessments of canagliflozin 100 and 300 mg as monotherapy [18], add-on to metformin [19], add-on to metformin plus sulfonylurea [20], and add-on to metformin plus pioglitazone [21]. In the add-on to metformin study, patients were randomized to receive canagliflozin 100 or 300 mg, sitagliptin 100 mg, or placebo once daily for 26 weeks; data from the sitagliptin arm were not included in this analysis. All studies in the pooled population enrolled men and women aged 18–80 years with T2DM. The monotherapy study included patients with HbA1c ≥7.0 and ≤10.0% and estimated glomerular filtration rate (eGFR) ≥50 mL/min/1.73 m^2^. The dual and triple therapy studies included patients with HbA1c ≥7.0 and ≤10.5% and eGFR ≥55 mL/min/1.73 m^2^. Patients were required to be on a stable antihypertensive medication regimen for ≥4 weeks prior to randomization; adjustments to antihypertensive medication considered clinically necessary were to be made during the pretreatment phase in order to avoid adjustments during the double-blind period. Key exclusion criteria common to all four studies included fasting plasma glucose (FPG) ≥15 mmol/L (≥270 mg/dL) during the pretreatment phase; history of type 1 diabetes mellitus (T1DM); history of myocardial infarction, unstable angina, revascularization procedure, or a cerebrovascular accident within 3 months of screening; and uncontrolled hypertension (i.e., the average of three seated BP readings with SBP ≥160 mmHg or DBP ≥100 mmHg). Details of the study design, including randomization and blinding, and glycemic rescue criteria have been previously reported for the individual studies included in the pooled analysis [18–21].

In the study evaluating canagliflozin in patients with T2DM and hypertension (N = 169) using 24-h ABPM [17], patients were randomized to receive canagliflozin 100 or 300 mg or placebo once daily for 6 weeks. The study included patients aged 18 to <75 years with T2DM and HbA1c ≥7.0 and <10.0% who were taking 1–3 AHAs, including metformin with or without sulfonylureas, thiazolidinediones, or dipeptidyl peptidase-4 inhibitors. Patients were also required to have hypertension (defined as a seated office SBP ≥130 and <160 mmHg and seated office DBP ≥70 mmHg) treated with stable doses of 1–3 antihypertensive agents, including either an angiotensin-converting enzyme (ACE) inhibitor or angiotensin II receptor blocker (ARB), with or without calcium channel blockers, β-blockers, or diuretics other than loop diuretics. Key exclusion criteria included a diagnosis of T1DM or diabetic ketoacidosis; repeated (i.e., ≥2 over a 1-week period) fasting self-monitored blood glucose measurements ≥13.3 mmol/L (240 mg/dL) during the pretreatment phase; uncontrolled hypertension (i.e., the average of three seated BP readings with SBP >160 mmHg or DBP >110 mmHg) at screening; treatment with an SGLT2 inhibitor, insulin, or a glucagon-like peptide-1 receptor agonist within 12 weeks prior to screening or in the 2-week run-in period; and treatment with antihypertensive therapy (i.e., ACE inhibitors, ARBs, loop diuretics, calcium channel blockers, or β-blockers) not on a stable regimen (i.e., same medications and doses) for ≥5 weeks prior to screening. Details of the study design, including randomization and blinding, have been previously reported [17].

All studies included in this analysis were conducted in accordance with ethical principles that comply with the Declaration of Helsinki, and were consistent with Good Clinical Practices and applicable regulatory requirements. Study protocols and amendments were approved by institutional review boards and independent ethics committees at participating institutions. All patients provided written informed consent prior to participation in the studies.

### Endpoints and assessments

Changes from baseline in SBP, DBP, pulse pressure, mean arterial pressure, and double product were assessed in the pooled, placebo-controlled studies at week 26 using seated BP measurements and in the ABPM study at week 6 using the averaged 24-h BP assessments. Pulse pressure was calculated as the difference between SBP and DBP. Mean arterial pressure was calculated as 2/3 DBP + 1/3 SBP. Double product was calculated as heart rate [beats per minute (bpm)] × SBP.

In the pooled, placebo-controlled studies, each seated BP measurement was based on an average of three BP readings taken manually with a mercury sphygmomanometer or an automated BP monitor at intervals of ≥1 min. In the ABPM study, BP recordings were collected over a 24-h period using an ABPM device every 20 min during the day and every 30 min during the night. Additional details regarding the methods used to measure BP have been previously reported for both study populations [16, 17].

Safety and tolerability were assessed based on adverse event (AE) reports through week 26 in the pooled, placebo-controlled studies and through week 6 in the ABPM study. AEs were reported spontaneously by patients or in response to non-directed questioning and were coded using the *Medical Dictionary for Regulatory Activities* (*MedDRA*). The overall incidence of AEs and the incidence of volume depletion–related AEs were evaluated.

### Statistical analyses

Analyses were performed using data from all randomized patients who received ≥1 dose of study drug. The last observation carried forward (LOCF) approach was used to impute missing data. Endpoints were analyzed using an analysis of covariance (ANCOVA) model, with treatment and stratification factor (ABPM study) or study (pooled, placebo-controlled studies) as fixed effects and the corresponding baseline value as a covariate. Least squares (LS) mean differences and 2-sided 95% confidence intervals (CIs) were estimated for the comparisons of each canagliflozin dose versus placebo. Safety analyses included all reported AEs (regardless of rescue therapy in the pooled, placebo-controlled studies), and included all randomized patients who received ≥1 dose of study drug. Statistical analyses were performed using SAS, version 9.2 (Cary, NC, USA).

## Results

### Patients

Baseline demographic and disease characteristics were generally similar across treatment groups in both study populations (Table 1). In the pooled, placebo-controlled studies, the mean baseline age was 56 years and patients had a mean baseline HbA1c of 8.0%, body mass index (BMI) of 32 kg/m^2^, and SBP of 128 mmHg. Of the 2313 patients included in the pooled studies, 1332 (57.6%) were taking antihypertensive medication at baseline. In the ABPM study, the mean baseline age was 59 years and patients had a mean baseline HbA1c of 8.1%, BMI of 33 kg/m^2^, and SBP of 139 mmHg. Mean baseline SBP and DBP were lower in the pooled, placebo-controlled studies, which included patients with and without hypertension, compared with the ABPM study, which enrolled only patients with hypertension.

**Table 1:** Baseline demographic and disease characteristics

	Pooled, PBO-controlled studies [16]			ABPM study [17]		
Characteristic^a^	PBO (n = 646)	CANA 100 mg (n = 833)	CANA 300 mg (n = 834)	PBO (n = 56)	CANA 100 mg (n = 57)	CANA 300 mg (n = 56)
Sex, n (%)						
Male	334 (52)	408 (49)	404 (48)	33 (59)	34 (60)	31 (55)
Female	312 (48)	425 (51)	430 (52)	23 (41)	23 (40)	25 (45)
Age, years	56.3 (9.8)	55.9 (10.1)	55.7 (9.5)	59.6 (9.5)	57.8 (8.7)	58.3 (6.9)
Race, n (%)^b^						
White	470 (73)	591 (71)	610 (73)	46 (82)	45 (79)	43 (77)
Black or African American	28 (4)	43 (5)	48 (6)	9 (16)	10 (18)	12 (21)
Asian	82 (13)	103 (12)	100 (12)	0	1 (2)	1 (2)
Other^c^	66 (10)	96 (12)	76 (9)	1 (2)	1 (2)	0
HbA1c, %	8.0 (0.9)	8.0 (0.9)	8.0 (1.0)	8.2 (0.9)	8.1 (0.9)	8.0 (0.8)
BMI, kg/m^2^	31.9 (6.4)	32.3 (6.4)	32.0 (6.5)	32.9 (5.7)	33.0 (6.0)	34.1 (6.8)
eGFR, mL/min/1.73 m^2^	87.0 (19.8)	88.3 (19.0)	88.8 (18.9)	87.9 (18.3)	87.2 (20.3)	85.6 (19.7)
Duration of T2DM, years	7.5 (6.2)	7.2 (5.8)	7.4 (6.2)	11.8 (8.7)	9.4 (6.0)	10.3 (6.2)
Seated SBP, mmHg	128.5 (13.2)	128.0 (12.8)	128.8 (12.8)	137.7 (8.6)	138.5 (11.1)	139.2 (8.8)
Seated DBP, mmHg	77.9 (8.3)	77.5 (8.0)	78.2 (8.3)	82.7 (8.6)	82.4 (7.7)	83.0 (8.2)

*ABPM* ambulatory blood pressure monitoring, *BMI* body mass index, *CANA* canagliflozin, *DBP* diastolic blood pressure, *eGFR* estimated glomerular filtration rate, *PBO* placebo, *SD* standard deviation, *SBP* systolic blood pressure, *T2DM* type 2 diabetes mellitus

^a^Data are mean (SD) unless otherwise indicated

^b^Percentages may not total 100% due to rounding

^c^Includes American Indian or Alaska Native, Native Hawaiian or other Pacific Islander, multiple, other, unknown, and not reported in the pooled, PBO-controlled studies; and includes other and unknown in the ABPM study

### Efficacy

#### Pooled, placebo-controlled studies

**Fig. 1:**
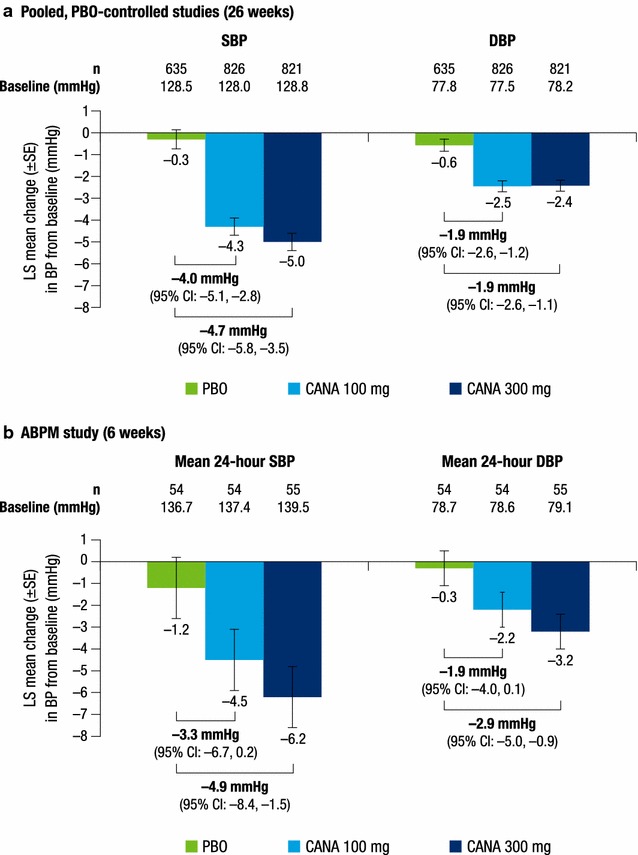
Change from baseline in **a** SBP and **b** DBP [16, 17]. *ABPM* ambulatory blood pressure monitoring, *CANA* canagliflozin, *CI* confidence interval, *DBP* diastolic blood pressure, *LS* least squares, *PBO* placebo, *SBP* systolic blood pressure, *SE* standard error. **a** was adapted from [16], with permission from John Wiley and Sons

In the pooled, placebo-controlled studies, canagliflozin 100 and 300 mg provided reductions in SBP and DBP compared with placebo at week 26 (Fig. 1a). LS mean changes from baseline in SBP with canagliflozin 100 and 300 mg and placebo were −4.3, −5.0, and −0.3 mmHg, respectively. LS mean changes from baseline in DBP with canagliflozin 100 and 300 mg and placebo were −2.5, −2.4, and −0.6 mmHg, respectively.

**Fig. 2:**
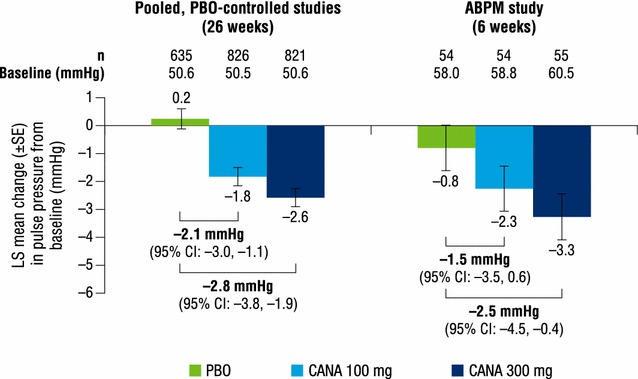
Change from baseline in pulse pressure. *ABPM* ambulatory blood pressure monitoring, *CANA* canagliflozin, *CI* confidence interval, *LS* least squares, *PBO* placebo, *SE* standard error

**Fig. 3:**
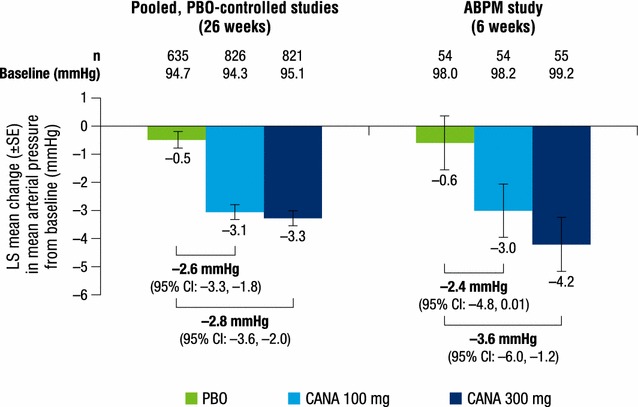
Change from baseline in mean arterial pressure. *ABPM* ambulatory blood pressure monitoring, *CANA* canagliflozin, *CI* confidence interval, *LS* least squares, *PBO* placebo, *SE* standard error

**Fig. 4:**
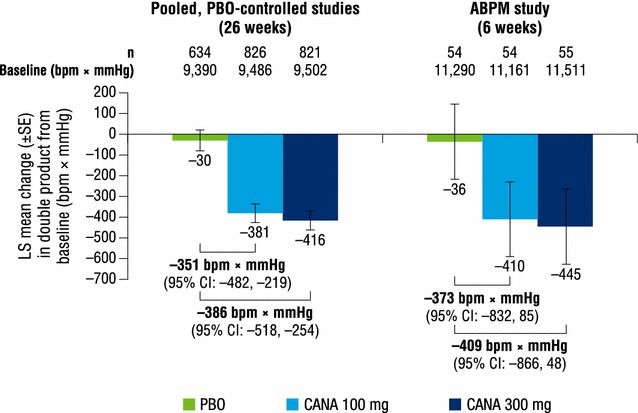
Change from baseline in double product. *ABPM* ambulatory blood pressure monitoring, *bpm* beats per minute, *CANA* canagliflozin, *CI* confidence interval, *LS* least squares, *PBO* placebo, *SE* standard error

Both canagliflozin doses reduced pulse pressure, mean arterial pressure, and double product compared with placebo at week 26 (Figs. 2, 3, 4). LS mean changes from baseline in pulse pressure were −1.8, −2.6, and 0.2 mmHg with canagliflozin 100 and 300 mg and placebo, respectively; LS mean changes in mean arterial pressure were −3.1, −3.3, and −0.5 mmHg, respectively. LS mean changes from baseline in double product were −381, −416, and −30 bpm × mmHg with canagliflozin 100 and 300 mg and placebo, respectively.

#### ABPM study

In the ABPM study, canagliflozin 100 and 300 mg were associated with reductions in mean 24-h SBP and DBP compared with placebo at week 6 (Fig. 1b). LS mean reductions from baseline in mean 24-h SBP were −4.5, −6.2, and −1.2 mmHg with canagliflozin 100 and 300 mg and placebo, respectively. LS mean changes in mean 24-h DBP were −2.2, −3.2, and −0.3 mmHg, respectively.

Dose-dependent reductions from baseline in pulse pressure were seen with canagliflozin 100 and 300 mg compared with placebo at week 6 (LS mean changes of −2.3, −3.3, and −0.8 mmHg, respectively; Fig. 2). Dose-dependent reductions were also seen in mean arterial pressure with both canagliflozin doses compared with placebo; LS mean changes from baseline were −3.0, −4.2, and −0.6 mmHg with canagliflozin 100 and 300 mg and placebo, respectively (Fig. 3). LS mean changes from baseline in double product were −410, −445, and −36 bpm × mmHg with canagliflozin 100 and 300 mg and placebo, respectively (Fig. 4).

### Safety

Canagliflozin 100 and 300 mg were generally well tolerated in the pooled, placebo-controlled studies and in the ABPM study, with low incidences of serious AEs and AEs related to study discontinuation across treatment groups in both studies [17, 22]. The tolerability profile of canagliflozin in both populations was generally consistent with previous studies, including a higher incidence of AEs related to the mechanism of SGLT2 inhibition (e.g., genital mycotic infections, osmotic diuresis–related AEs).

In the pooled, placebo-controlled studies, rates of volume depletion–related AEs (e.g., hypotension, postural dizziness, orthostatic hypotension) were low across groups (1.2, 1.3, and 1.1% with canagliflozin 100 and 300 mg and placebo, respectively). Among patients treated with canagliflozin, no volume depletion–related AEs were considered serious or led to study discontinuation. In the ABPM study, 2 patients (3.6%) experienced a volume depletion–related AE with canagliflozin 300 mg; none were reported with canagliflozin 100 mg or placebo. Rates of significant orthostasis [defined as symptoms on standing (e.g., dizziness, lightheadedness) or a reduction in office SBP ≥20 mmHg or DBP ≥15 mmHg 2 min after standing] at week 6 were 3.8, 7.1, and 3.9%, with canagliflozin 100 and 300 mg and placebo, respectively.

## Discussion

In the post hoc analysis of pooled data from four Phase 3 studies, canagliflozin 100 and 300 mg provided reductions in SBP and DBP, as well as pulse pressure, mean arterial pressure, and double product compared with placebo over 26 weeks in a broad population of patients with T2DM. Responses were generally similar in the post hoc analysis of data from a smaller, 6-week ABPM study in patients with T2DM and hypertension. Both canagliflozin doses reduced mean 24-h SBP and DBP over 6 weeks compared with placebo. Canagliflozin 300 mg provided reductions in pulse pressure and mean arterial pressure compared with placebo, while canagliflozin 100 mg had more modest effects on these parameters. In both study populations, canagliflozin was generally well tolerated, with an increase in AEs related to the mechanism of SGLT2 inhibition (e.g., osmotic diuresis–related AEs), consistent with previous Phase 3 studies of canagliflozin [15].

The findings from this analysis are consistent with those reported for the SGLT2 inhibitor empagliflozin in a study that evaluated changes in BP and markers of arterial stiffness and vascular resistance in patients with T2DM [23]. Empagliflozin provided significant reductions in SBP and DBP, pulse pressure, mean arterial pressure, and double product in a post hoc analysis of pooled 24-week data from four Phase 3 studies in patients with T2DM. Similar improvements were seen in a post hoc analysis of data from a 12-week ABPM study of empagliflozin in patients with T2DM and hypertension [23]. Collectively, the results from the canagliflozin and empagliflozin studies suggest that improvements in arterial stiffness and vascular resistance may be class effects of SGLT2 inhibitors. Results from the current analysis demonstrated a reduction in BP and associated parameters within 6 weeks of initiating canagliflozin that persisted through 26 weeks of treatment. Earlier responses on BP with other SGLT2 inhibitors have not been well characterized, but in the canagliflozin ABPM study, small numeric improvements in 24-h SBP were noted after 1 day of treatment. Of note, canagliflozin has been shown to provide a reduction in plasma volume within 1 week due to increased UGE and natriuresis, which may contribute to initial BP lowering in patients with T2DM [24].

The favorable effects of canagliflozin treatment on pulse pressure, mean arterial pressure, and double product are expected to translate into reduced arterial stiffness, improved blood flow, and a lower cardiac workload. Furthermore, as these markers have been shown to be predictive of cardiovascular events [8, 9, 25–27], the improvements in BP seen with canagliflozin treatment, in addition to reductions in HbA1c and body weight, may contribute to better cardiovascular outcomes in patients with T2DM. In the EMPA-REG OUTCOME trial, empagliflozin showed a significant reduction in the risk for major adverse cardiovascular events, cardiovascular death, and hospitalization for heart failure compared with placebo in patients with T2DM and established cardiovascular disease [28]. It is not yet known whether the improved cardiovascular outcomes observed with empagliflozin apply to the entire SGLT2 inhibitor class, but recent meta-analyses support favorable cardiovascular outcomes with SGLT2 inhibitors [29–34]. It has been hypothesized that the increase in osmotic diuresis associated with SGLT2 inhibition results in a lower BP and intravascular volume that may help reduce cardiac workload [35, 36]; however, further studies are needed to define the cardioprotective mechanisms of SGLT2 inhibition. Results from the ongoing CANagliflozin cardioVascular Assessment Study (CANVAS; ClinicalTrials.gov Identifier: NCT01032629 [37]) and CANVAS-R (renal endpoints; NCT01989754 [38]) trials in patients with a history or high risk for cardiovascular disease are expected in 2017 and will help determine whether the improvements in cardiovascular outcomes seen with empagliflozin are representative of the SGLT2 inhibitor class.

Limitations of the study include the post hoc nature of the analysis and the relatively small number of patients in the ABPM study population. In addition, double product may be best assessed using ABPM and many not accurately predict cardiovascular risk in some patient populations [39]. However, the use of various measures in two different studies showing concordant results in two different study populations representing a broad range of patients with T2DM, including those with hypertension, strengthens the analysis.

## Conclusions

Canagliflozin provided reductions in pulse pressure, mean arterial pressure, and double product over 26 weeks in a general population of patients with T2DM and in a 6-week ABPM study in patients with T2DM and hypertension. The improvements in cardiovascular physiologic markers seen with canagliflozin in this analysis are consistent with the hypothesis that canagliflozin may have beneficial effects on some cardiovascular outcomes in patients with T2DM. Results from the ongoing CANVAS and CANVAS-R trials may help to better define the cardioprotective effects of canagliflozin in patients with T2DM.

## Abbreviations

ABPM: ambulatory blood pressure monitoring
ACE: angiotensin-converting enzyme
AE: adverse event
AHA: antihyperglycemic agent
ANCOVA: analysis of covariance
ARB: angiotensin II receptor blocker
BMI: body mass index
BP: blood pressure
bpm: beats per minute
CANA: canagliflozin
CANVAS: CANagliflozin cardioVascular Assessment Study
CANVAS-R: CANagliflozin cardioVascular Assessment Study–Renal
CI: confidence interval
DBP: diastolic BP
eGFR: estimated glomerular filtration rate
FPG: fasting plasma glucose
LOCF: last observation carried forward
LS: least squares
PBO: placebo
SBP: systolic BP
SGLT2: sodium glucose co-transporter 2
SE: standard error
T1DM: type 1 diabetes mellitus
T2DM: type 2 diabetes mellitus
UGE: urinary glucose excretion
